# Specific Features of SVZ Neurogenesis After Cortical Ischemia: a Longitudinal Study

**DOI:** 10.1038/s41598-017-16109-7

**Published:** 2017-11-27

**Authors:** S. Palma-Tortosa, A. García-Culebras, A. Moraga, O. Hurtado, A. Perez-Ruiz, V. Durán-Laforet, J. de la Parra, M. I. Cuartero, J. M. Pradillo, M. A. Moro, I. Lizasoain

**Affiliations:** 1 Unidad de Investigación Neurovascular, Departamento de Farmacología and Instituto Universitario de Investigación en Neuroquímica, Facultad de Medicina, Universidad Complutense; Instituto de Investigación Hospital 12 Octubre (i+12), Madrid, Spain; 2 Present Address: James Black Centre, Cardiovascular Division, King’s College London BHF Centre, London, United Kingdom

## Abstract

Stroke is a devastating disease with an increasing prevalence. Part of the current development in stroke therapy is focused in the chronic phase, where neurorepair mechanisms such as neurogenesis, are involved. In the adult brain, one of the regions where neurogenesis takes place is the subventricular zone (SVZ) of the lateral ventricles. Given the possibility to develop pharmacological therapies to stimulate this process, we have performed a longitudinal analysis of neurogenesis in a model of cortical ischemia in mice. Our results show an initial decrease of SVZ proliferation at 24 h, followed by a recovery leading to an increase at 14d and a second decrease 28d after stroke. Coinciding with the 24 h proliferation decrease, an increase in the eutopic neuroblast migration towards the olfactory bulb was observed. The analysis of the neuroblast ectopic migration from the SVZ toward the lesion showed an increase in this process from day 14 after the insult. Finally, our data revealed an increased number of new cortical neurons in the peri-infarct cortex 65d after the insult. In summary, we report here critical check-points about post-stroke neurogenesis after cortical infarcts, important for the pharmacological modulation of this process in stroke patients.

## Introduction

Stroke is one of the main causes of death and disability in the adulthood in developed countries and leads to huge socioeconomic costs. While part of the current research is focused on limiting ischemic damage in the initial stages after the insult, many efforts are currently devoted to investigate the mechanisms that underpin brain repair following injury, in an attempt to develop strategies that enhance reparative endogenous processes, such as adult neurogenesis. In contrast with Ramon y Cajal’s arguments about the non-regenerative properties of the adult nerve system, the existence of adult neurogenesis has been demonstrated in the mammalian brain. Under physiological conditions this process is spatially restricted to two specific neurogenic brain niches: the subventricular zone (SVZ) of the lateral ventricles and the subgranular zone (SGZ) in the dentate gyrus of the hippocampus^1^. While neurogenesis in the SGZ has been mainly related to memory and learning, in the SVZ neural stem cells proliferate and generate neuroblasts which migrate along the rostral migratory stream (RMS) to the olfactory bulb (OB), where they differentiate to new neurons and integrate into the neuronal circuitry^2^. In pathological situations such as an ischemic stroke, there is an increase in the proliferation of these neuronal precursors, mostly at the SVZ, that migrate to the lesion site and differentiate to functional neurons around the infarct^3,4^.

Post-stroke neurogenesis has been widely studied in experimental models with striatal affectation, such as the intraluminal middle cerebral artery occlusion (MCAO) in rodents, which show a clear time course of the different steps of neurogenesis (proliferation and neuroblast migration) with the final appearance of new neurons in the damaged striatum^3,5^. However, very few have performed a longitudinal exploration of neurogenesis after infarcts limited to the cortical region. In the first studies where cortical ischemia was induced by the intraluminal model causing both, striatal and cortical damage, no significant numbers of new neurons were found in the ischemic cortex^3,5^. In contrast, in later works using specific cortical stroke models, such as the photothrombotic one and the distal occlusion of the middle cerebral artery, the presence of new neurons in the peri-infarct cortex was demonstrated^6–8^. However, as regards cortical neurogenesis, the specific time course of the different steps of this process and their duration is not yet clear due to variations in the model used and the pattern, location, extend and dynamics of the cortical ischemic lesions. It has been also demonstrated that cortical post-stroke neurogenesis can be enhanced by additional manipulation (i.e. growth factor infusion or acute inhibition of inflammation)^9–11^.

Therefore, since a detailed study is lacking of endogenous neurogenesis-induced after cortical injury and considering the interest of new therapeutic targets for repair in chronic stroke, in this study we aimed to analyze the temporal profile of proliferation, migration and survival of new neuroblasts and their differentiation to mature neurons from the SVZ to the damaged cortex in a model of cortical ischemia in mice.

## Results

### Infarct volume and SVZ proliferation after permanent cerebral ischemia

**Figure 1:**
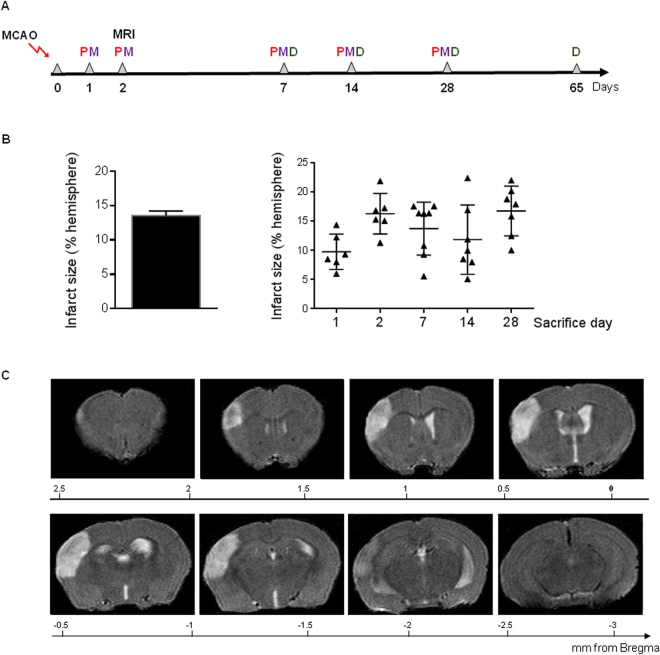
(**A**) Diagram indicating days of sacrifice and MRI when proliferation (P), migration (M) and neuronal differentiation (D) were studied. (**B**) Percentage of infarcted hemisphere 48 hours after experimental ischemia. (**C**) Representative T_2_W images acquired from an ischemic brain 48 h after MCAO. Data were compared by non-parametric 1-way ANOVA followed by Bonferroni post-hoc testing (p > 0.05).

In order to obtain similar infarct volumes in all the animals and avoid the influence of the lesion size on SVZ cell proliferation as demonstrated previously^10^, all the surgeries were made by the same investigator. As shown in Fig. 1(B,C), percentage of infarct volume was 13.6 ± 0.64 with no differences in infarct size among all the animals used in this study (p > 0.05).

**Figure 2:**
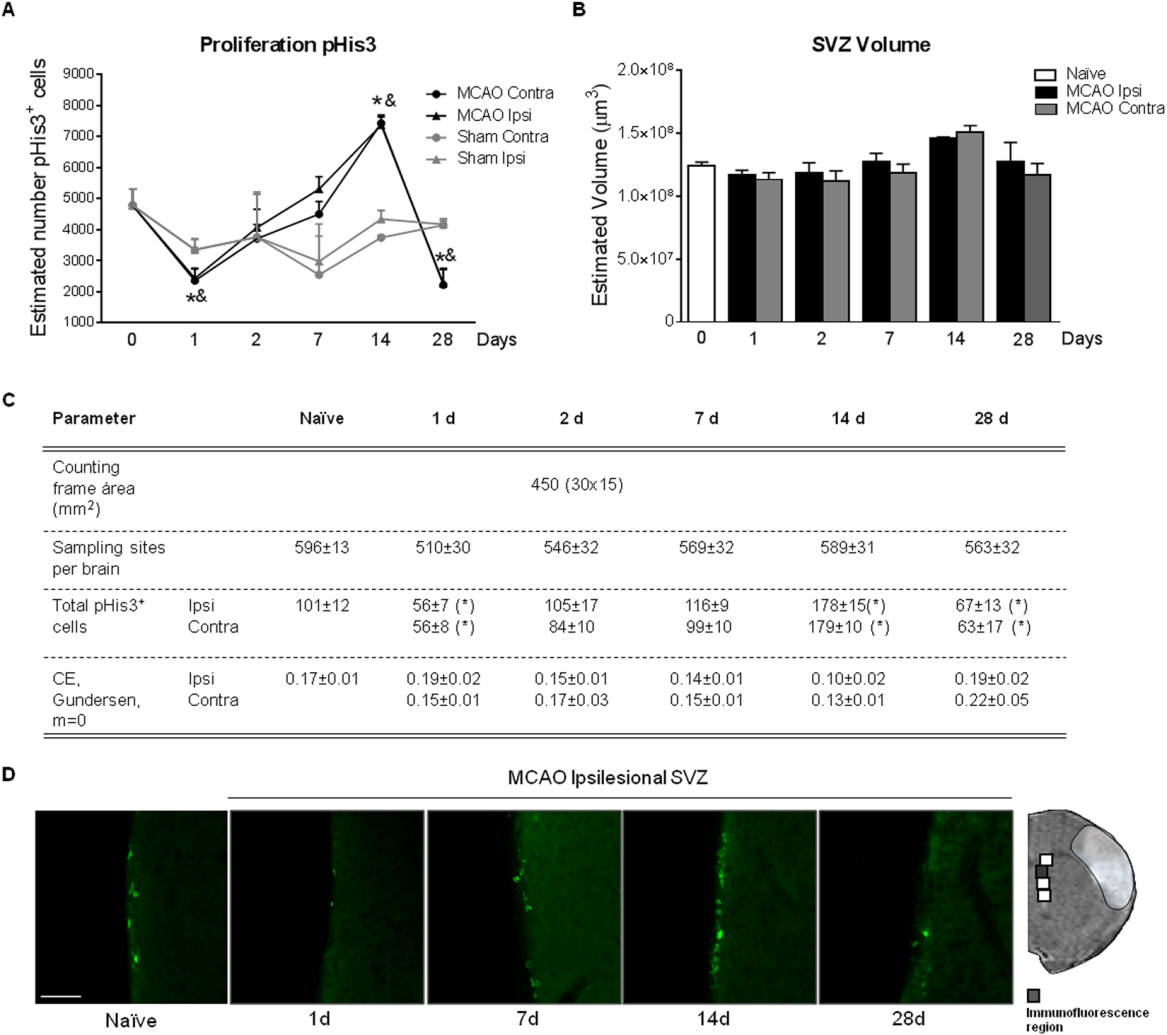
Effect of stroke on SVZ proliferation analyzed by pHis3^+^ immunofluorescence in (**A**) MCAO (n = 6–8), sham (n = 4) and naïve (n = 8) animals. (**B**) Estimated SVZ volume calculated by Cavalieri estimator. (**C**) Parameters used for the stereological quantification of pHis3^+^ cells. (**D**) Representative images of pHis3 immunohistochemistry of naïve and ischemic groups at different time points after experimental ischemia (scale bar: 50 µm). Naïve group was represented in figure as t = 0. No differences were observed when naïve and sham animals were compared. Data are expressed as mean +/− SEM and compared by non-parametric 2-way ANOVA followed by Bonferroni post-hoc testing (F(1,38) = 12.26, *p < 0.0012, Naïve vs. MCAO 1d; F(1,20) = 7.642, ^&^p = 0.0120, Sham vs. MCAO 1d; F(1,32) = 17.32; *p = 0.0002, Naïve vs. MCAO 14d; F(1,10) = 170.1, ^&^p < 0.0001, Sham vs. MCAO 14d; F(1,38) = 12.21; *p = 0.0012, Naïve vs. MCAO 28d; F(1,16) = 12.01, ^&^p = 0.0032, Sham vs. MCAO 28d).

Our first objective was to determine the proliferative status of the SVZ neurogenic niche in an experimental model of cortical cerebral ischemia. Our results show a bilateral and triphasic response in the number of proliferating (pHis3^+^ cells) in the SVZ after cortical stroke, characterized by first, a significant decrease 1d post-injury (p < 0.05, MCAO 1d vs. Naïve; p < 0.05, MCAO 1d vs. Sham 1d), secondly, a gradual recovery leading to a significant increase at 14d (p < 0.05, MCAO 14d vs. Naïve; p < 0.05, MCAO 14d vs. Sham 14d) and finally, a second reduction at 28d (p < 0.05, MCAO 28d vs. Naïve; p < 0.05, MCAO 28d vs. Sham 28d) in the two hemispheres, when compared with SVZ proliferation basal levels (Fig. 2A). Analysis of the SVZ volume in these experimental groups did not show any differences at any post-stroke time points (Fig. 2B; p > 0.05). No differences were observed either in SVZ proliferation when naïve and sham animals were compared (data not shown).

### Longitudinal profile of eutopic neuroblast migration from SVZ to OB after cortical ischemic injury

**Figure 3:**
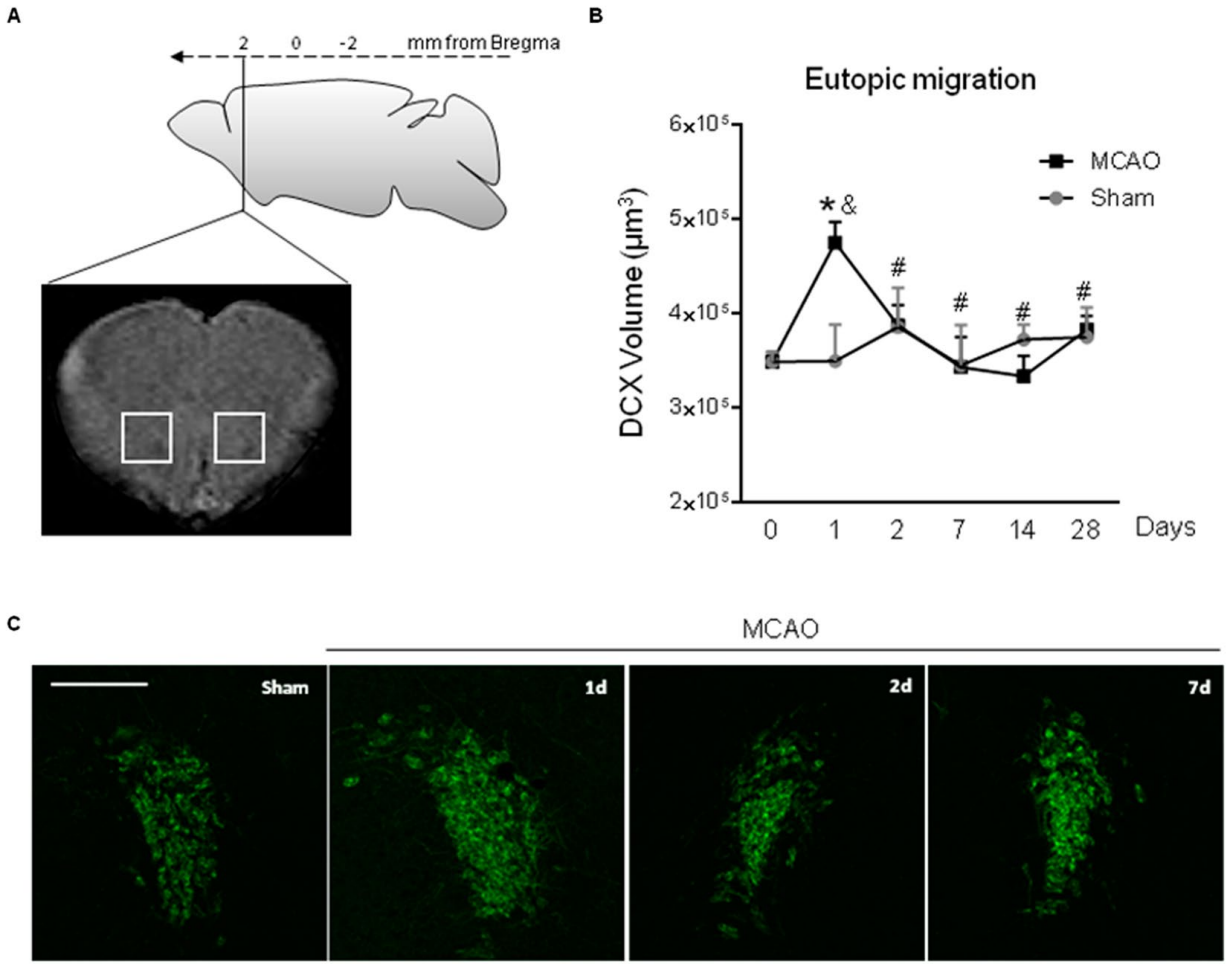
Temporal profile of eutopic neuroblast migration after stroke. (**A**) Image showing the brain region analyzed. (**B**) DCX^+^ staining volume and (**C**) representative images from each experimental group (scale bar: 100 µm) were represented. Naïve group was represented in figure as t = 0. No differences were observed when naïve and sham animals were compared. Data are expressed as mean +/− SEM and compared by non-parametric 1-way ANOVA followed by Dunnett post-hoc testing, *p = 0.0012 vs. Naïve and ^#^p < 0.005 vs. MCAO 1d. Non-parametric 2-tailed Mann-Whitney t-test was used to compare MCAO 1d vs. Sham (^&^p = 0.035).

In order to investigate whether the normal neuroblast migration route from the SVZ towards the OB through the RMS was affected by cortical ischemia, an analysis of the amount of migrating neuroblasts in this region was performed at different time points after stroke (Fig. 3A). Our results show an increased neuroblast migration to the OB at 24 h after the cortical injury (p < 0.05, MCAO 1d vs. Naïve; p < 0.05 MCAO 1d vs. Sham 1d) followed by a return to basal levels from day 2 up to day 28 (Fig. 3B,C, p < 0.05, MCAO vs. MCAO 1d). No differences were found between naïve and sham groups at any of the time points studied (p > 0.05, Sham vs. Naïve).

### Longitudinal profile of the ectopic neuroblast migration from the SVZ to the ischemic cortex

**Figure 4:**
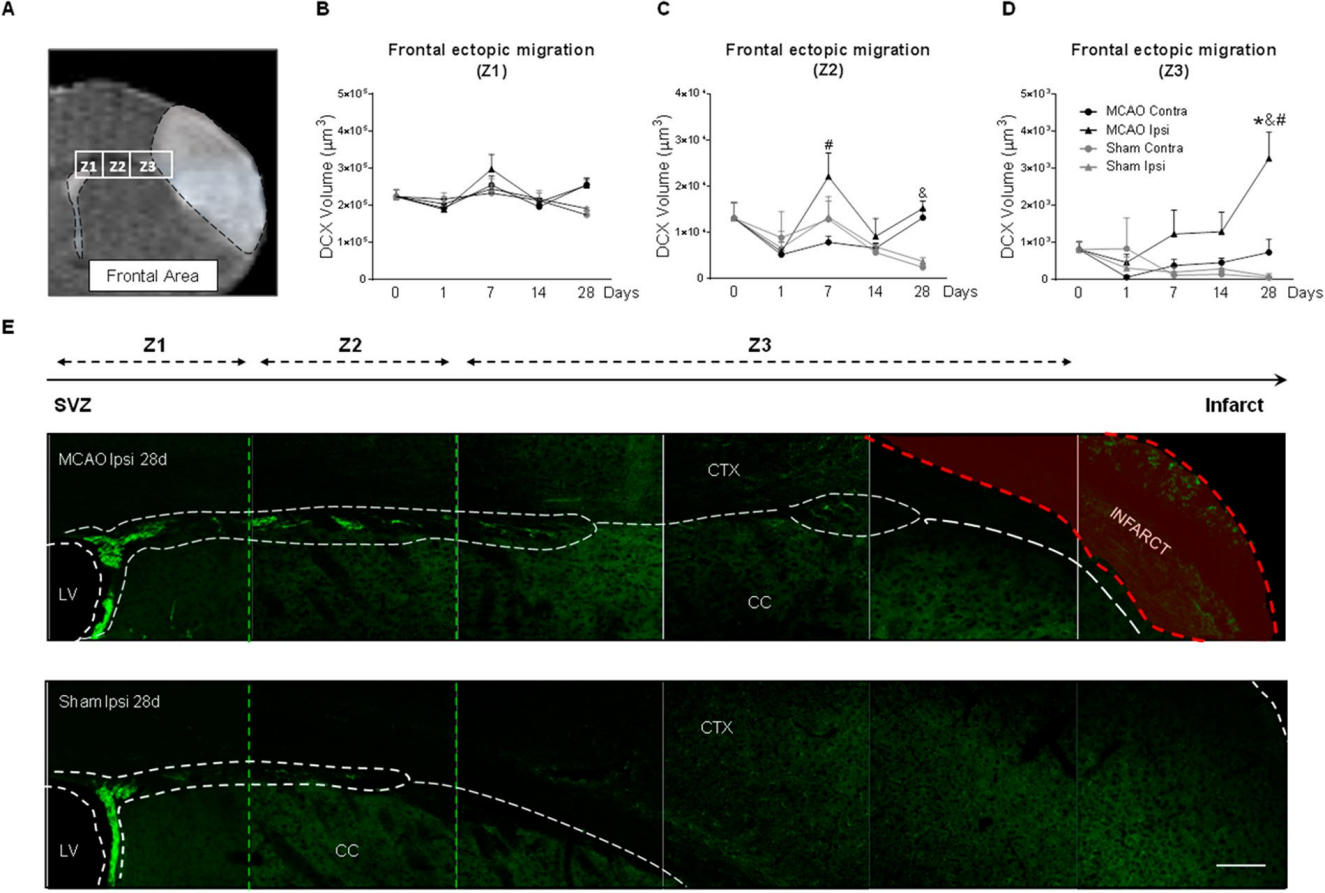
Frontal ectopic neuroblast migration after stroke. (**A**) Regions analyzed from the SVZ toward the infarct (Z1, Z2, Z3). DCX^+^ volume in (**B**) Z1, (**C**) Z2 and (**D**) Z3. (**E**) Representative neuroblast migration images in MCAO and sham animals at 28d after stroke (scale bar: 100 µm). Naïve group was represented in figure as t = 0. No differences were observed when naïve and sham animals were compared. Data are presented as mean +/− SEM and compared by non-parametric 2-way ANOVA followed by Bonferroni post-hoc testing (Z2: F(1,14) = 2.94, ^#^p = 0.0246, Ipsi MCAO vs. Contra MCAO; F(1,14) = 36.08, &p < 0.0001, MCAO vs. Sham; Z3: F(1,14) = 4.96, ^#^p = 0.0429 Ipsi MCAO vs. Contra MCAO; F(1,14) = 11.15, ^&^p = 0.0049 MCAO vs. Sham) and non-parametric 1-way ANOVA followed by Dunnett post-hoc testing (Z3: *p = 0.0099 MCAO vs. Naïve).

**Figure 5:**
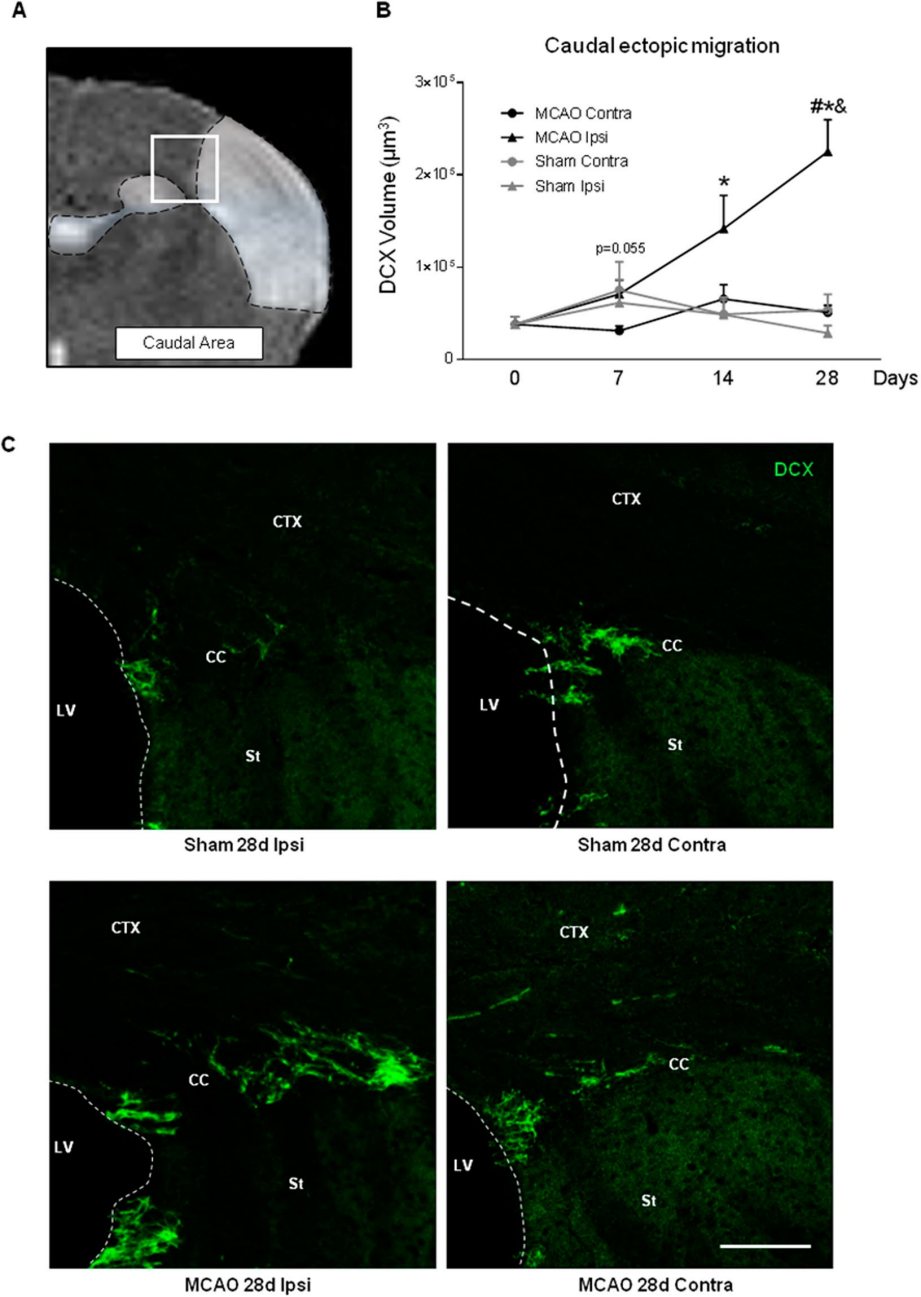
Caudal ectopic neuroblast migration after stroke. (**A**) Region analyzed from the SVZ to the infarct. (**B**) DCX^+^ staining volume. (**C**) Representative neuroblast migration images in MCAO and sham animals at 28d after stroke (scale bar: 100 µm). Naïve group was represented in figure as t = 0. No differences were observed when naïve and sham animals were compared. Data are expressed as mean +/− SEM and compared by non-parametric 2-way ANOVA followed by Bonferroni post-hoc testing (F(1,13) = 10.16, ^#^p = 0.0071 MCAO Ipsi 28d vs. Contra 28d, F(1,13) = 17,20, ^&^p = 0.0011 MCAO 28d vs. Sham 28d) and non-parametric 1-way ANOVA followed by Dunnett post-hoc testing (*p = 0.0225 MCAO 14d vs. Naïve, *p = 0.0002 MCAO 28d vs. Naïve).

We then asked how and when a cortical ischemic injury modifies neuroblast migration route (*ectopic migration*) by analyzing the presence of neuroblasts in two brain areas to cover the extension of the infarct (Figs 4A and 5A). Regarding the frontal ectopic route, three zones, Z1-3, were studied, from Z1 (nearest region to the SVZ) to Z3 (closest region to infarct). No differences were detected in the amount of migrating neuroblasts between both hemispheres at any of the post-stroke time points analyzed in Z1 (Fig. 4B, p > 0.05, MCAO vs. Naïve; p > 0.05, MCAO vs. Sham). In Z2, although no differences on the number of migrating neuroblasts on post-stroke day 7 versus naïve/sham animals were found, a significant increase in this ectopic migration was observed in the ipsilateral hemisphere compared with the contralateral hemisphere (p < 0.05, MCAO Ipsi vs. MCAO Contra). Also, an increase in neuroblast migration was found 28 days after ischemia in MCAO animals (both at ipsi- and contralesional hemispheres) compared to sham animals (Fig. 4C, p < 0.05, MCAO vs. Sham). Finally, in region Z3, there was a significant increase in the number of migrating neuroblasts at 28d after the injury compared with sham animals and the contralateral hemisphere (Fig. 4D,E, p < 0.05, MCAO vs. Sham; p < 0.05 Ipsi vs. Contra; p < 0.05, MCAO vs. Naïve). In this frontal region, no differences in neuroblast migration were found at any of the times studied and in any of the regions analyzed between naïve and sham animals (p > 0.05).

Regarding the caudal area, our results show a unilateral, ectopic migration of neuroblasts from the SVZ to the lesion site after stroke from day 7 (p = 0.055) with a significant increase in the ipsilateral hemisphere at days 14 and 28 compared with non ischemic animals (Fig. 5B,C, p < 0.05, Ipsi vs. Contra; p < 0.05, MCAO vs. Naïve; p < 0.05, MCAO vs. Sham).

### Neuronal differentiation/incorporation to the peri-infarct cortex

**Figure 6:**
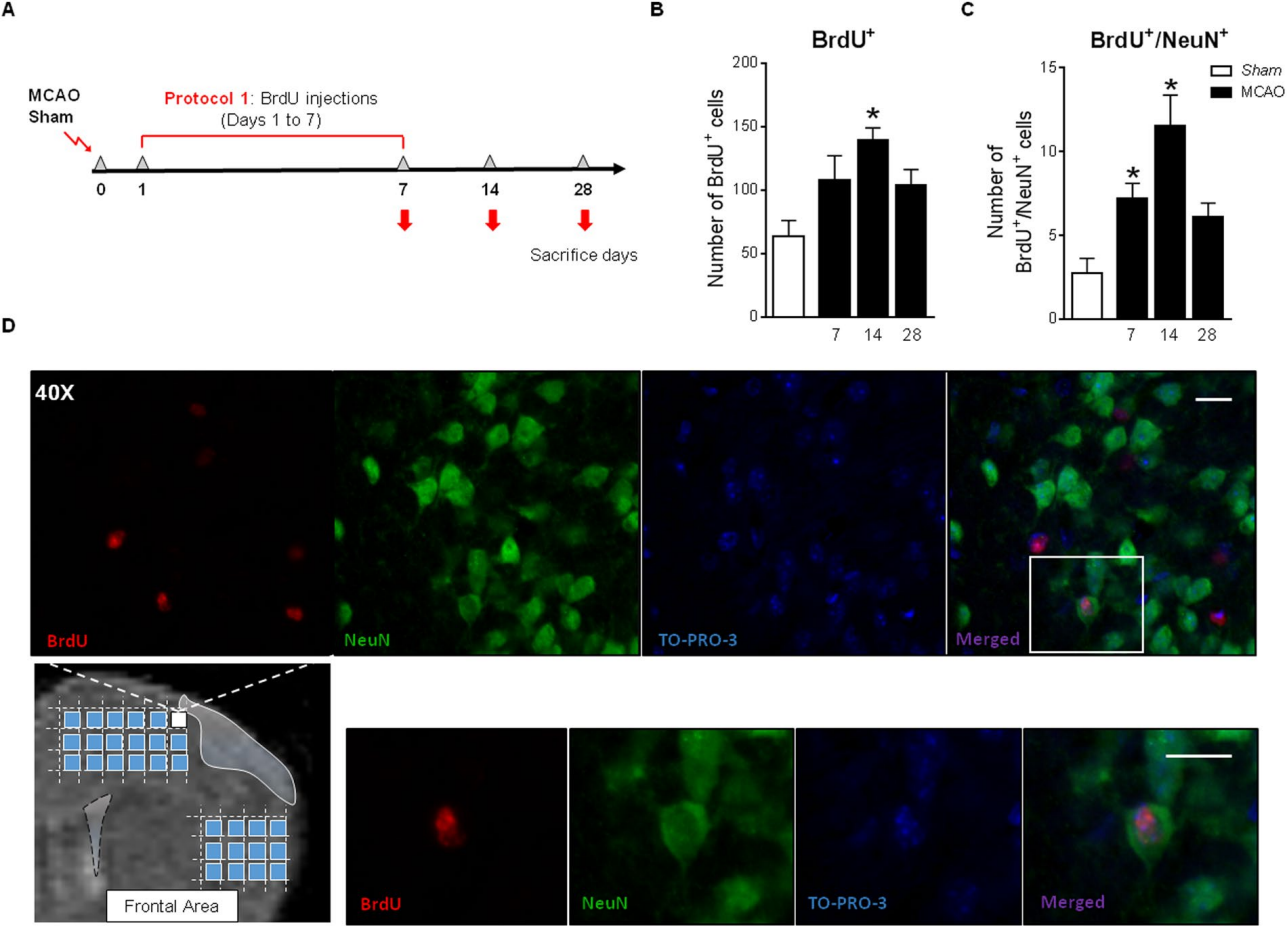
(**A**) Neuronal differentiation after protocol 1 of BrdU administration. (**B**) Number of BrdU^+^ cells and (**C**) new neurons (BrdU^+^/NeuN^+^ cells) in the ipsilesional cortex from 7-28d after MCAO. (**D**) Brain image showing the cortical regions analyzed and representative images of a BrdU^+^/NeuN^+^/TO-PRO-3^+^ cell (scale bar: 25 µm). Sham mice represented in Fig. 6B and C were sacrificed at 7 days, and did not show any differences with 14d and 28d sham. Data are expressed as mean +/− SEM (n = 5–8). Non-parametric 1-way ANOVA followed by Dunnett post-hoc testing were used to analyze data (BrdU^+^: *p = 0.0421 MCAO vs. Sham; BrdU^+^/NeuN^+^: *p = 0.0011 MCAO vs. Sham).

We finally explored the appearance of newborn neurons in the peri-infarct cortex after cortical ischemia in mice. For that purpose, the administration of BrdU, a proliferation marker, was performed following two different protocols: first, corresponding with the recovery of proliferation from days 1 to 7 after stroke in the SVZ, and second, coinciding with the proliferation peak from 14 to 28d after the injury. Since a similar number of BrdU and BrdU/NeuN positive cells was observed in sham animals sacrificed at 7, 14 and 28 days (data not shown) with both protocols, the sham values represented in Fig. 6B,C correspond to those of the sham-7d experimental group. Following protocol 1 (Fig. 6A), our results revealed an enhanced number of BrdU^+^ cells in the frontal peri-infarct cortex at day 14 after the injury, when compared with the sham group (Fig. 6B, p < 0.05 MCAO vs. Sham). Among these cells, we observed an increase in the number of newborn neurons (BrdU^+^/NeuN^+^ cells) in the peri-infarct area at days 7 and 14 after cortical injury versus sham group (Fig. 6C, p < 0.05, MCAO vs. Sham). However, a reduction was found in the post-stroke day 28. TO-PRO-3 immunostaining was performed in order to verify the nuclear position of the BrdU staining (Fig. 6D).

**Figure 7:**
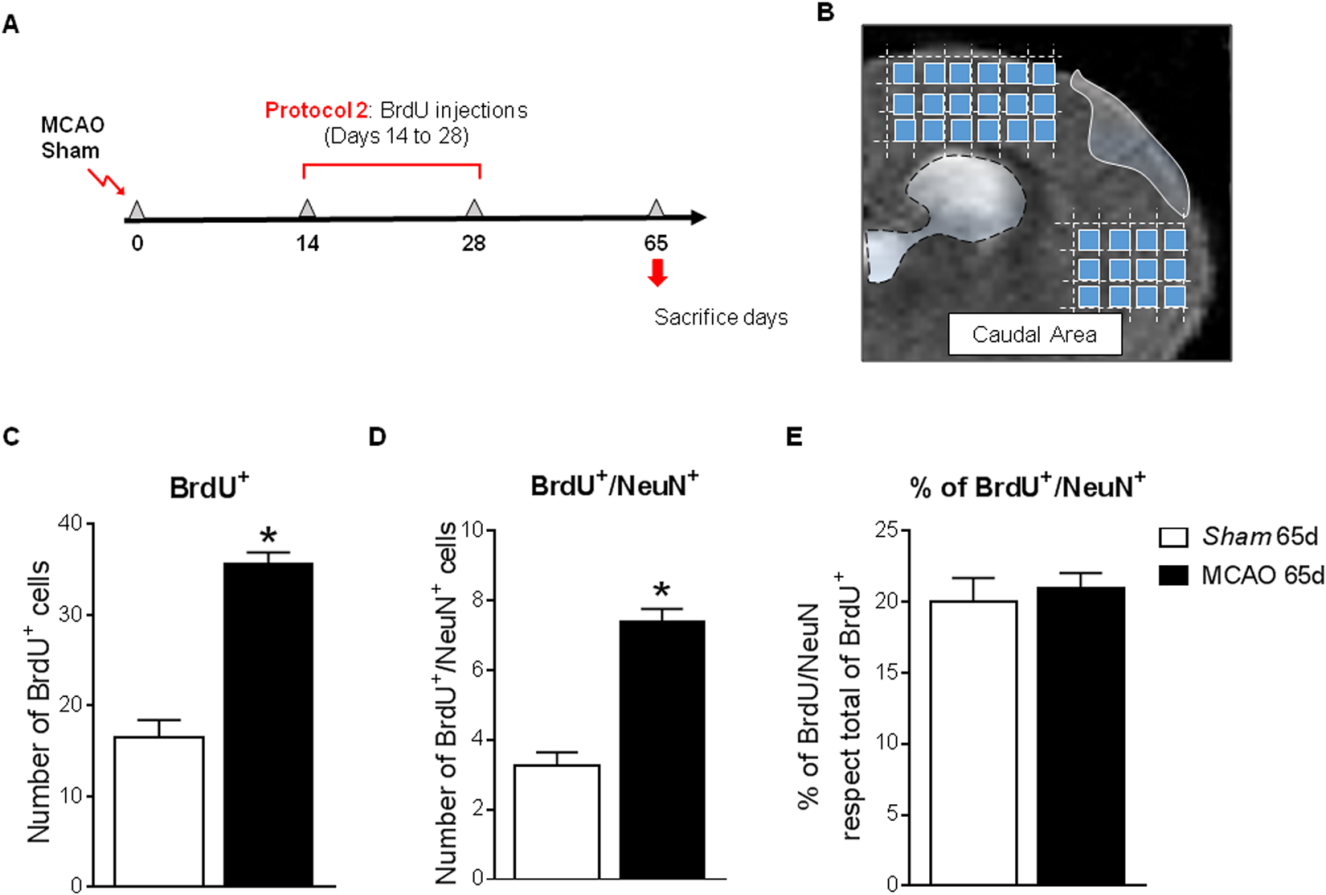
(**A**) Neuronal differentiation after protocol 2 of BrdU administration and (**B**) brain image showing the cortical regions analyzed. (**C**) Number of BrdU^+^ cells and (**D**) newborn neurons (BrdU^+^/NeuN^+^ cells) in the ipsilesional cortex 65d after MCAO. (**E**) Neuronal cell fate. Data expressed as mean +/− SEM. Non-parametric 2-tailed Mann-Whitney t-test was used to compare data (*p = 0.0286 MCAO vs. Sham, n = 5–8).

On the other hand, with protocol 2 (Fig. 7A,B), our data show an increase in BrdU^+^ cells in the caudal peri-infarcted cortex compared to sham animals at 65d after the injury (Fig. 7C, p < 0.05, MCAO vs. Sham); among these cells, the number of newborn neurons (BrdU^+^/NeuN^+^ cells) was also higher in the peri-infarct of ischemic animals versus sham (Fig. 7D, p < 0.05, MCAO vs. Sham).

Finally, we studied whether cortical ischemia changes the cell fate of the SVZ proliferative cells between days 14 to 28 after stroke. The percentage of newborn neurons (NeuN^+^) among the total number of BrdU^+^ cells in the peri-infarct cortex, approx. 20%, revealed no differences between ischemic and sham animals (Fig. 7E, p > 0.05).

## Discussion

Under certain pathological conditions such as brain ischemia, the brain is able to produce new neurons theoretically aimed to regenerate the damaged region and to improve the neurological functions (for review, see refs^4,12,13^). However, whereas most studies have shown an increase in neurogenesis after striatal ischemia^3,5,14–16^, only a few have explored specifically the effects of cortical cerebral ischemia^10,17,18^ and, among them, no one has performed a systematic description of the process. Therefore, in an attempt to understand better how cortical ischemia affects this neurorepair process, we have performed a longitudinal study of the overall proliferative status of the SVZ, the migration of neuroblasts and their differentiation to mature neurons in the damaged cortex in a model of cortical cerebral ischemia in mice.

First, we have found that cortical stroke has a triphasic effect on the total number of cells in proliferation at the SVZ, first with an early acute reduction of proliferation on post-stroke day 1, a second slow increase with a maximum on post-stroke day 14 and finally, a reduction of proliferating cells at 28 days after cortical ischemia. Interestingly, these changes were only observed in ischemic animals, while naïve and sham animals did not show any changes on proliferation during time. Our results also show that this process is bilateral in all the conditions and at all the time points studied, supporting an important effect of ventricular cerebrospinal fluid (CSF) composition at this level. This bilaterality in the SVZ was previously described after cerebral ischemia by intraluminal MCAO occlusion^19^.

At each time point, the number of proliferating cells present in the niche results from the balance between proliferation and migration. Regarding the early acute decrease in the number of proliferating cells at the SVZ, it could be argued that the proliferation process is reduced at this time and/or that the cells migrate away at a higher rate from this area. In addition, to our knowledge, our data are the first to describe that cortical stroke induces an increased neuroblast migration through their physiological RMS route acutely after stroke (day 1), supporting that such increased migration towards the OB accounts, at least in part, for the decrease in the number of cells in proliferation at the SVZ at the same time.

Whereas the majority of studies have assessed post-stroke SVZ proliferation during the sub-chronic phase (7 to 10 days) and using BrdU as a proliferation marker in order to obtain information of past and accumulative proliferation^20,21^, in our study we have analyzed the SVZ proliferation also at early time-points by quantification of pHis3^+^ cells, that correspond to cells in mitosis at the moment of the animal sacrifice.

From 2 to 7 days after cortical ischemia, our results show a recovery of SVZ proliferation, with a significant increase at 14 days, followed by a reduction 28 days after the insult. Other authors have also described a proliferation peak between days 7 to 10 after a striatal damage using the intraluminal stroke model^3,19^ or embolus placement in rat^22,23^ and also in humans around day 10 after stroke^24,25^.

Interestingly, we have observed a second reduction in SVZ proliferation at 28 days vs. basal levels. Peaks of proliferation in the SGZ have been shown to be followed by a decrease in cell division and neurogenesis^26,27^ as a result of an exhaustion of the niche, an effect that may also explain our results.

Although we have not explored later times, other studies demonstrate a return of proliferation to basal levels around the 6^th^ week after the induction of cerebral ischemia^14^, an effect that is likely to occur also in our model.

We have additionally studied neuroblast migration after cortical ischemia. We have analyzed both the physiological RMS route (from the SVZ to the OB; eutopic migration) and also the occurrence of an ectopic diversion from this route towards the damaged area. First, as commented above, our results reveal a significant increase in eutopic migration only 24 hours after the insult, with a recovery to basal levels from 2 to 28 days after stroke. As discussed above, this enhanced migration might explain the early reduction in the number of proliferating cells at the SVZ during the first hours after the insult. Although further studies are required to clarify the mechanisms involved, changes in CSF composition after stroke are likely to account for this process.

On the other hand, we have analyzed neuroblast migration from the SVZ to the ischemic cortex (ectopic migration), a process described after stroke in several animals models^3,28,29^ and human studies^24,30^. In our study, we have examined a long frontal area and a shorter caudal area. Our results show an increase in neuroblast migration near the lesion at post-stroke days 14 and 28 at the caudal level, but only at 28 days in the frontal one, very likely due to the longer distance in the latter.

In contrast with the effects induced by stroke on the proliferation in the SVZ, which are bilateral at all the different times studied, migration is unilateral. This supports the idea that, while variations in CSF composition are capable of inducing changes in SVZ proliferation as well as migration in the RMS in the acute phase (24 hours), the ectopic migration of the neuroblasts, driven only in the ipsilateral zone, is due to cues originating from the infarcted and/or peri-infarct area, as already demonstrated^31–34^.

In this context, several mechanisms involved in these processes, although still not completely elucidated, have been widely reported in the literature. For instance, previous studies carried out by several groups including ours have identified different pathways/mediators that regulate the proliferation process in the SVZ such as Notch signaling^35^, miRNAs levels^36^ and the TNF/TNFR-1 axis^37^ and we have already demonstrated the participation of different proteins such as TLR4 and CB2R receptors^10,11,38^. It has been also demonstrated that cerebral ischemia increases the cortical expression of VEGF, BDNF, SDF-1α and Ang1, molecules that induce changes in the vasculature and induce the glial tube to help the migration of neuroblasts to the ischemic lesion^31,39,40^.

Theoretically, the final fate of injury-induced neurogenesis is to replace dead neurons and/or to provide a favorable environment by delivering newborn neurons to the damaged area. Our results indicate that cells that proliferate during the first week after ischemia (cells labeled at post-stroke days 1–7), despite successfully differentiating into neuroblasts and reaching the ischemic cortex, are not able to survive long term. This may be because the number of cells that proliferate at this time is lower and/or due to the ischemic environment at that time, still hostile due to the inflammatory processes activated after brain damage, impeding the integration process^41^. Although the possible direct functional benefit of this limited neurogenesis is doubtful, it is possible that these neuroblasts that reach the injured area contribute to the functional recovery through the contribution of trophic factors and/or other substances that help the remaining cells to solve the conflict in which they are. In contrast, the study of the cells proliferating between days 14 to 28 (corresponding with the proliferation peak described) shows a remarkable increase in the number of new neurons integrated in the cortex at 65 days after the injury, indicating that cerebral ischemia induces an effective neurogenesis that translates into the appearance of new neurons in the ipsilesional cortex.

The ratio of new neurons generated to the total number of marked cells reaching the cortex is 20%, corresponding to that described in the literature^42^, for both sham and MCAO groups, supporting that cerebral ischemia, at least in our model, does not modify the percentage of cells destined to become new neurons.

In spite of the fact that the final number of mature neurons integrated in the infarcted cortex seems to be very low, different strategies such as exercise, environmental and enrichment, reduction of the acute post-stroke inflammation/oxidative stress and infusion of mesenchymal/human derived stem cells have been proposed to be able to increase this process. However, up to date, the exact moment in which the application of these strategies could maximize the process of neurogenesis and improve the stroke outcome is still not clear, highlighting the importance of knowing the temporal course of the different phases of this neurogenic process.

In conclusion, we report a descriptive study of the longitudinal profile of neurogenesis in the SVZ of mice subjected to cortical injury by a permanent MCAO, which shows the importance of both SVZ proliferation and neuroblast migration to deliver new neurons to the peri-infarct cortex. Our data provides novel descriptive information and opens new lines of investigation on important checkpoints to be targeted to develop specific therapies that could increase neurogenesis and improve the outcome of stroke patients.

## Materials and Methods

### Animals

2–3 month-old male C57BL/6 mice (Jackson Laboratory, Bar Harbor, ME, USA) were used for this study. Mice were housed under standard conditions of temperature, ventilation and humidity with a 12-h light-dark cycle (lights on at 08:00) with free access to food and water. All the experimental procedures were performed under the evaluation of the Animal Welfare Committee of the Universidad Complutense (ES280790000086) and the Consejería de Medio Ambiente y Ordenación del Territorio de la Comunidad de Madrid (under the Spanish Royal Law RD 53/2013) following the European directives 86/609/CEE and 2003/65/CE.

### Experimental groups, permanent focal ischemia and BrdU administration

All animals were randomly allocated to the following experimental groups: naïve (n = 8 per time point), sham (n = 4 per time point) and ischemic group (MCAO; n = 6–8 per time point) and all the analyses were performed and quantified by investigators blinded to each specific treatment (Fig. 1). Permanent focal ischemia was performed in C57BL/6 mice following the model described by Chen *et al*.^43^ with slight modifications. Briefly, all the surgical procedure was conducted under anesthesia with isoflurane 3% for induction and 1.5% for maintenance in a mix of O_2_ and N_2_O (0.2/0.8 L/min) and the temperature was maintained at 37.0 ± 0.5 °C using a servo-controlled rectal probe-heating pad. Cerebral ischemia was performed by a permanent ligation of the trunk of the left middle cerebral artery (MCAO) with a 9–0 suture, in combination with the occlusion of the ipsilateral common carotid artery^44^. Animals where the common carotid and MCA were exposed but not occluded were considered as sham animals.

To determine the number of new integrated neurons at different time points after the injury, two protocols of bromodeoxyuridine (BrdU; Sigma; UK) administration were used. The first one (protocol 1; Fig. 6A) consisted on the intraperitoneal injection of this agent at a dose of 50 mg/kg/daily, from days 1 to 7 after stroke, to analyze at 7,14 and 28d the neuronal fate of cells that proliferate during the first week of the insult. In the second one (protocol 2; Fig. 7A), BrdU was also administered intraperitoneally at a dose of 100 mg/kg, 5d per week, from days 14 to 28 after the injury, to analyze at 65d after stroke the neuronal fate of cells that proliferate during BrdU administration (Fig. 7A).

### Infarct size determination

Infarct size was determined on T_2_W brain images acquired in a BioSpec BMT 47/40 magnet (Bruker, Ettlingen, Germany). These brain images were acquired 2 days after MCAO in all the experimental groups, with the exception of the animals sacrificed 1d after the injury, in which the T_2_W images were obtained 21 hours after experimental ischemia. The analysis of the images was performed using ImageJ 1.44 l (U.S. National Institutes of Health, Bethesda, MD, USA). The ipsilateral (A^Ips^) and contralateral hemispheres (A^Contr^) and the infarcted tissue (A^Inf^) areas were delineated in 12 equidistant images separated by 500 µm, quantifying a total of 6 mm in the rostro-caudal axis from 2.5 to −3.5 mm of bregma. Then, the infarct size was calculated and expressed as percentage of the ipsilesional hemisphere (%IH) as previously described^45^, using the following formula %IH = V^Inf^/V^Contr^ × 100, where V^Inf^ = ∑A^Inf^-ipsilateral/SI-ipsilateral, being SI swelling index, V^Contr^ = ∑A^Contr^i, and SIi = A^Ips^i/A^Contr^i.

### Tissue processing

Mice were transcardially perfused at different time points (1, 2, 7, 14, 28 and 65 days) after MCAO with 0.1 M phosphate buffer (pH 7.4) followed by 4% p-formaldehyde (PFA; pH 7.4). Brains were removed, post-fixed overnight in 4% PFA, placed in 30% sucrose for 48 h and frozen at −80 °C. Brain sections (40 µm) were cut on a freezing microtome (Leica SM2000R; Leica Microsystems GmbH, Wetzlar, Germany) and stored at −20 °C in antifreeze solution (30% ethylene glycol and 20% glycerol in phosphate-buffered saline) until processing.

### Immunofluorescence staining

With this technique, we evaluated different markers of neurogenesis. We first analyzed the overall SVZ cell proliferation on days 1, 2, 7, 14 and 28 after naïve/sham or stroke surgical procedures by staining for pHis-3, a mitosis marker. Immunofluorescence was performed on free-floating sections and incubated overnight at 4 °C with the primary antibody rabbit anti-P-His (1:300; ABD Serotec, Bio-Rad Laboratories Inc., Hercules CA, USA). Then, sections were washed and incubated during 2 h with the fluorescent secondary antibody goat anti-rabbit biotin (Vector Laboratories, Peterborough, UK) in combination with Alexa-488 streptavidin (Molecular Probes; Life Techologies, Madrid, Spain).

For the study of neuroblast migration from the SVZ to the OB through the RMS (*eutopic migration route*) and to the lesion site at different longitudinal brain levels (*ectopic routes*), sections were stained for doublecortin (DCX), a microtubule associated phosphoprotein used as a neuroblast marker. This immunofluorescence was performed as described above, on days 1, 7, 14 and 28 after sham and MCAO by incubating the sections with the primary antibody goat anti-DCX (1:200; Santa Cruz Biotechnology, Heidelberg, Germany) followed by the fluorescent secondary antibody horse anti-goat biotin (1:200, Vector Laboratories, Peterborough, UK) and Alexa488 streptavidin.

Finally, neuronal differentiation was analyzed at 7, 14, 28 and 65d after ischemia by NeuN^+^/BrdU^+^ cells quantification. We used also TO-PRO-3 to verify the nuclear position of BrdU. To label the cell nuclei, sections were incubated first with TO-PRO-3 (1:10000; Thermofisher Scientific, UK) during 15 min, fixed for 15 min in 4% paraformaldheyde solution, and washed with PBS. Then to visualize BrdU, sections were first incubated in HCl 2 N at 37 °C for 30 min in order to denature the DNA chain and, after washing in PBS and blocking the non-specific bindings, sections were incubated with the primary antibodies anti-neuronal nuclei (NeuN; 1:200; MAB 377; Millipore, Billerica MA, USA) and rat anti-BrdU (1:200; ABD Serotec, Bio-Rad Laboratories Inc., Hercules CA, USA) followed by horse anti-mouse biotin, donkey Cy3 anti-rat (1:200, Jackson Immunoresearch, Suffolk, UK) and Alexa488 streptavidin secondary antibodies.

Negative controls performed in parallel without primary antibodies showed very low levels of non-specific staining.

### Unbiased stereology

The total volume of the dorsolateral extension of the SVZ was estimated by application of the Cavalieri principle using Cavalieri estimator on 5 serial sections per brain (40 µm thickness, 0.32 mm apart; bregma 1.70 to 0.54 mm). The morphological criteria used for the delineation of the SVZ are described in Gonzalez *et al*.^46^. Stereological estimation of the total number of pHis-3^+^ cells was performed in the selected area using the optical fractionator method^47^. The specific parameters used for stereological sampling and quantification are summarized in Fig. 2C.

### Cell quantification on confocal images

The analysis of the neuroblast migration was studied at different levels after cortical stroke. First, we studied the normal migratory route from the SVZ to the OB through the RMS (*eutopic* route; Fig. 3A). Second, we analyzed the changes towards an abnormal route from the SVZ to the lesion site through the corpus callosum (*ectopic* route; Figs 4A and 5A). As our ischemic model causes a wide infarct along the cortex (from -2,5 to 2,5 mm of bregma), the ectopi*c* neuroblast migration was analyzed in two brain areas: a long, frontal area, close to the RMS (*frontal ectopic migration*), and a shorter, caudal area, along the extension of the ischemic lesion (*caudal ectopic migration*). Images of DCX-immunofluorescence staining were acquired in a blinded manner by laser-scanning confocal microscopy (LSM710; Zeiss, Munich, Germany) at 20x with 14-µm thickness and in *z-stack format*. For the RMS study, the analysis was performed on images acquired from 2 consecutive sections spaced 320 µm between them and starting at 2 mm from bregma. For the analysis of the frontal ectopic migration (from the SVZ to the lesion site through the *corpus callosum*), z-stack images were acquired from 5 consecutive sections, starting at 1.70 mm until 0.1 mm from bregma. On each section, a first image of the SVZ was obtained (Z1), a second adjacent image (Z2) along the corpus callosum was also acquired, and finally, two or three consecutive images were acquired (region Z3) to fully analyze the migration of the neuroblasts towards the infarct area, following a protocol previously described^10^. Finally, to study the caudal ectopic migration (from the caudal part of the SVZ to the lesion site), z-stack images were acquired from 3 consecutive sections separated by 320 µm, starting in bregma (0) until −0.96 mm from this point. The analysis of all DCX-images was performed by integrated density (DInt) calculations using Volocity 3D image analysis software (Perkin-Elmer, USA).

For the determination of the number of newborn mature neurons (BrdU^+^/NeuN^+^ cells), immunofluorescence images acquired by confocal microscopy were taken from 5 consecutive sections beginning at 1.20 mm from bregma until 0.02 mm for the study with BrdU protocol 1, and from bregma until −1.6 mm for the BrdU protocol 2. The images were obtained at 40x, spaced 800 µm from each other to cover the entire cortex around the core of the lesion, using as boundaries the corpus callosum and the end of the cortex. A total of 18–20 images/ipsilateral- hemisphere/section were obtained, and the quantification of the new neurons was made using ZEN 2009 software (Zeiss). All colocalisation images shown were confirmed by orthogonal projection of the z-stack files.

### Statistical analysis

Results are expressed as mean ± standard error of the mean (SEM). Unpaired Student’s t test was used to compare 2 groups or one-way or 2-way ANOVA to compare more than 2 groups with Dunnett or Bonferroni post hoc tests correspondingly. Differences were considered significant at p < 0.05.
